# Curving expectations: The minimal impact of structural curvature in biological puncture mechanics

**DOI:** 10.1126/sciadv.adp8157

**Published:** 2024-08-14

**Authors:** Bingyang Zhang, Bishal Baskota, Jules J. Chabain, Philip S. L. Anderson

**Affiliations:** Department of Evolution, Ecology, and Behavior, School of Integrative Biology, University of Illinois Urbana-Champaign, 505 S. Goodwin Ave., Urbana 61801, IL, USA.

## Abstract

Living organisms have evolved various biological puncture tools, such as fangs, stingers, and claws, for prey capture, defense, and other critical biological functions. These tools exhibit diverse morphologies, including a wide range of structural curvatures, from straight cactus spines to crescent-shaped talons found in raptors. While the influence of such curvature on the strength of the tool has been explored, its biomechanical role in puncture performance remains untested. Here, we investigate the effect of curvature on puncture mechanics by integrating experiments with finite element simulations. Our findings reveal that within a wide biologically relevant range, structural curvature has a minimal impact on key metrics of damage initiation or the energies required for deep penetration in isotropic and homogeneous target materials. This unexpected result improves our understanding of the biomechanical pressures driving the morphological diversity of curved puncture tools and provides fundamental insights into the crucial roles of curvature in the biomechanical functions of living puncture systems.

## Teaser

Curvature in biological puncture tools like talons and fangs does not affect their ability to penetrate tissues.

## INTRODUCTION

**Fig. 1.**
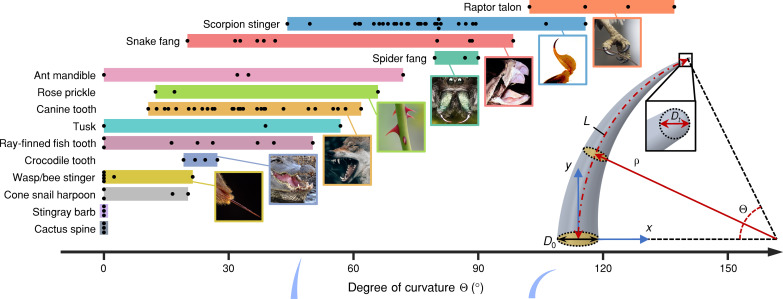
The diversity and geometry of curved biological puncture tools. A spectrum of curvature (characterized by degree of curvature Θ) spans different types of selected biological puncture tools across taxa. Black dots indicate data from different species (see table S2 for the entire list of measurements and sources). Bottom right: A puncture tool is modeled as a curved cone with a centerline arc of length *L* and radius of curvature ρ spanning angle Θ = *L*/ρ in the *x*-*y* plane. The diameter of a circular cross section perpendicular to the centerline diminishes linearly from *D*_0_ at the base to *D_t_* at the tip. Image credit (from top to bottom): Red-tailed hawk talons (*Buteo jamaicensis*): adapted from russimages, flickr.com (CC BY 2.0); Scorpion stinger: adapted from dw_ross, flickr.com (CC BY 2.0); Viper fangs (*Bitis gabonica*): adapted from Brimac The second, flickr.com (CC BY 2.0); Spider fangs (*Phidippus johnsoni*): Kaldari, wikimedia.org (public domain); Rose prickles: adapted from Ingrid Taylar, flickr.com (CC BY 2.0); Wolf canine teeth: Philipp Pilz, unsplash.com (public domain); American alligator teeth (*Alligator mississippiensis*): adapted from W. L. Farr, wikimedia.org (CC BY 4.0); Wasp stinger (*Polistes carolina*): insects unlocked, flickr.com (public domain).

Biological puncture mechanisms, ranging from venom-injecting viper fangs and scorpion stingers, to herbivore-deterring rose prickles, to prey-capturing claws, talons, and canine teeth, serve critical functional roles in the survival strategies of diverse organisms across the tree of life (*1*–*14*). Underpinning these complex functionalities is the remarkable morphological diversity of biological puncture tools. While most tools exhibit a tapered, sharp-tipped shape, they can vary substantially in sizes (*7*, *10*, *14*), sharpness (*7*, *9*, *12*), ornamentations (*8*, *15*–*18*), and material architectures (*4*, *19*, *20*). In particular, structural curvature of biological puncture tools across phyla ranges widely from a nearly linear shape found in stingray barbs and cactus spines (degree of curvature, Θ ≈ 0°) to a highly curved crescent shape found in raptor (bird of prey) talons (degree of curvature, Θ ≳ 100°), with scorpion singers, snake fangs, spider fangs, canine teeth, and other puncture tools exhibiting intermediate curvatures (as illustrated in Fig. 1).

Despite the wide variety of curved puncture tools across a broad range of taxa, the biomechanical pressures driving their morphological diversity remain unclear. Structural curvature of natural puncture tools is hypothesized to play various important biomechanical roles, both during the two distinct phases of puncture—damage initiation and deep penetration—and in other functions beyond puncture. Experimental and computational studies of structural biomechanics and load-bearing capabilities associated with variation in curvature have suggested that curved puncture tools may offer superior resistance to buckling and structural damage under prescribed bending loads caused by indentation and scratching during damage initiation compared to their linear counterparts (*5*, *6*, *16*, *21*). It was reported that this effect might be further enhanced by nonhomogeneous material composition, structural gradients, and hierarchical architectures of puncture tools, as observed in deep-penetrating, venom-injecting scorpion stingers (*4*, *22*) and spider fangs (*19*, *22*–*25*). Beyond mechanical strength and damage resilience, curved tools are also theorized to benefit certain biological functions. For example, boas use curved teeth located at different positions in their jaws to align with head rotation occurring during strikes as well as prey grasping, and swallowing (*11*), similar to how predatory insects (*26*) and fish-hunting spearing mantis shrimps (*7*, *27*) use curved claws and appendages for effective predation. Broadly, curved tools across biological scales also support and facilitate gripping, pinching, climbing, perching, foraging, and other crucial functional activities (*1*, *3*, *7*, *26*, *28*–*31*). Many of these functionalities uniquely rely on a curved shape and are much more challenging to attain via linear tools.

**Fig. 2.**
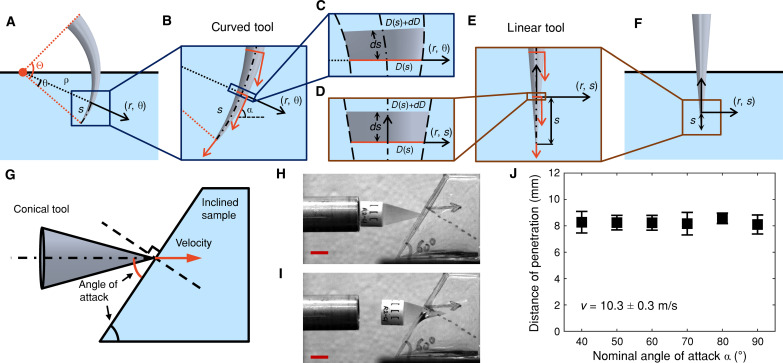
Deep penetration is independent of attack angle in isotropic and homogeneous target materials. (**A** to **F**) Schematic illustrations comparing the deep penetration configurations of curved and linear puncture tools. *s* denotes the distance between the tool tip and the cross section of interest along the centerline. A local polar coordinate system (*r,*θ) and a local Cartesian coordinate system (*r,s*) are established at *s* for curved and linear tools, respectively. The orange arrows in (B) and (C) indicate velocity vectors at different tool locations, which define the local angle of attack α. The infinitesimal thin slices taken at *s* are mathematically identical between curved (C) and linear (D) tools as *ds* → 0, with the same cross-sectional diameter *D*(*s*) = *D*_0_*s*/(ρΘ). (**G**) Schematic illustration of the dynamic puncture test with an inclined target surface. The angle of attack is controlled by the inclined angle of the target material. (**H** and **I**) Still-frame high-speed images captured at the onset of impact and maximum penetration, respectively. Scale bars, 10 mm. (**J**) Measurements of the maximum distance of penetration from inclined samples do not depend on the applied nominal angle of attack (*P* = 0*.*97, one-way ANOVA) within a range of α = 40° to 90° at a puncture velocity of *v* = 10*.*3 ± 0*.*3 m/s. Each error bar represents the mean and SD calculated from three replicates.

While extensive research has explored the roles of curvature in the strength and biological functions of puncture tools, there is a gap in the literature on how variation in structural curvature can affect puncture mechanics. Understanding potential trade-offs in puncture performance/efficiency and other biomechanical performance of various curved puncture tools will offer fundamental insights into their functional morphology and the evolutionary biomechanics of living puncture systems. While the existence of such trade-offs was theorized by Bar-On (*21*) based on bending/load-bearing characteristics of curved tools, systematic verification requires incorporating tool-material interactions during puncture. To this end, a few studies in biomedical engineering have examined the insertion forces of curved biomedical needles (e.g., suture needles and steerable needles) in biological tissues (*32*–*34*) and reported a minor influence of curvature (*33*). However, the geometries and materials of these biomedical tools are markedly different from those of natural puncture tools. To our knowledge, the effect of curvature of natural puncture tools on key biomechanical metrics, such as forces and energies required for puncturing biological materials, has yet to be investigated and quantified. Evolutionarily, a substantial effect of curvature comparable to the effect of tool sharpness [e.g., (*9*, *35*)] would likely result in limited morphological variation in structural curvature to attain optimal puncture performance. However, the broad diversity of puncture tool curvatures observed in nature suggests otherwise (e.g., Fig. 1). Therefore, we propose a null hypothesis based on local tool-material interactions and the energetics of deep penetration (Fig. 2) that states that, within a biologically relevant range, structural curvature is not a substantial biomechanical factor that influences puncture performance. We aim to test this null hypothesis through a combination of puncture experiments and computational simulations, with a focus on quantification of the effect of curvature on both initiation and deep penetration phases of puncture. In contrast to linear counterparts, curved puncture tools exhibit unique configurations and dynamics of tool-material interaction in natural puncture scenarios, including rotational movement and a circular penetration path (*4*, *11*). Understanding the mechanics of such nonlinear failure geometry will not only inform the potential drivers of biological puncture tool diversity but also guide the design of bio-inspired tools in biomedical engineering and other applications.

## RESULTS

### A theory for the independence of curvature

**Fig. 3.**
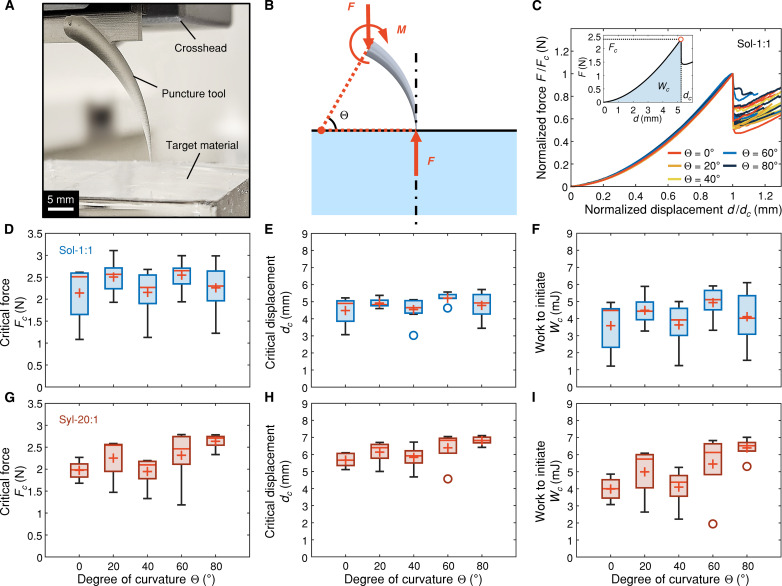
Effect of curvature on damage initiation. (**A**) Image of a damage initiation test with a 3D printed tool of Θ = 60°. (**B**) Schematic illustration of the configuration of damage initiation. The puncture tool tip is positioned perpendicular to the target material surface. The force applied by the crosshead and the reaction force at the tip are eccentric, resulting in a finite moment of *M*. (**C**) Normalized force versus displacement responses for damage initiation tests of Sol-1:1 PDMS samples show coinciding elastic indentation regimes across all replicates within Θ = 0° to 80°. Force (*F*) and displacement (*d*) data are normalized by the corresponding critical values (*F_c_* and *d_c_*) at damage initiation. Inset: A representative force versus displacement profile obtained at Θ = 60°, illustrating the critical force *F_c_* and displacement *d_c_* extracted at the critical point of damage initiation/force drop (open red circle), and work to initiate *W_c_* calculated from the area under the curve up to *d_c_*. (**D** to **I**) Box-and-whisker plots summarize the results of critical characteristics across Θ = 0° to 80°. Each data point was calculated from at least five replicates, with the mean (orange cross), median (orange line), and interquartile range included. (D) to (F) and (G) to (I) present measurements from Sol-1:1 and Syl-20:1 PDMS target materials, respectively. *F* values and *P* values from one-way ANOVA are provided in table S1.

At the initiation of puncture, the critical load and deformation state are governed by a combination of intrinsic ultimate properties of the target material and the contact mechanics between the tool tip and material surface, which is largely independent of the tool curvature. We test this hypothesis in Fig. 3 by conducting damage initiation tests with various curved tools. In contrast, the deep penetration phase, characterized by fracture propagation and strain energy release driven by the unique rotational movement of the curved puncture tool, requires a distinct consideration. Here, we incorporate an established puncture energetics model and propose a theoretical framework—accounting for changes in the orientation and angle of attack of a curved tool in an ideal deep penetration scenario—to provide insights into the mechanics underlying the curvature independence of puncture.

**Fig. 4.**
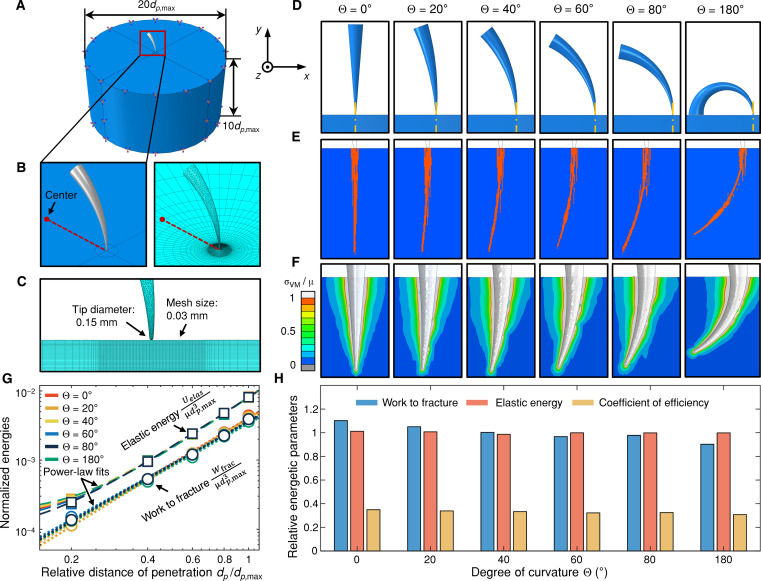
Effect of curvature on deep penetration. (**A** to **C**) The dimensions and mesh size of a representative FE model (Θ = 60°) in different views of the initial configuration. The target material substrate implements “encastre” boundary conditions on the side and bottom surfaces. The undeformed damage/fracture surface (orange shaded region) (**E**) and the shear modulus normalized von Mises stress field (σ_VM_/μ) (**F**) corresponding to the maximum distance of penetration (*d*_*p,*max_ = 5 mm) are compared across different prescribed tool curvatures (Θ = [0*,* 20*,* 40*,* 60*,* 80*,* 180]°) (**D**). (**G**) Scaling relationships between normalized energies and distance of penetration *d_p_*/*d*_*p,*max_, and the corresponding power-law fits obtained across different curvatures. (**H**) Relative magnitudes of energetic parameters (elastic energy, work to fracture, and coefficient of efficiency) at *d*_*p,*max_ plotted as a function of Θ.

Figure 2 illustrates and compares two-dimensional representations of ideal curved and linear puncture tools during deep penetration into a target material, modeled as a half-space comprising isotropic and homogeneous soft materials. Both puncture tools have the same base diameter and taper linearly to an extremely sharp tip (*D_t_* ≈ 0). They are perpendicular to the target material surface and have identical distances of penetration, *d_p_*, along their centerlines. Similar to the finite element (FE) model (Fig. 4), the curved puncture tool adopts a circular path concentric with its centerline. We assume an ideal biological puncture system exhibiting (i) ductile failure, with the deformed fracture surface conforming to the tool surface during penetration, and (ii) negligible deflection/indentation at the target material surface, consistent with the failure behavior of our silicone tissue simulants and FE model (*35*–*37*). In this case, the total energy required for deep penetration of a linear puncture tool can be estimated using the following established scaling relationship (*36*)

$$W\left(d_{p}\right)=W_{\text{frac}}+U_{\text{elas}}+W_{\text{fric}}=k_{\text{frac}}\Gamma d_{p}^{2}+k_{\text{elas}}\mu d_{p}^{3}+k_{\text{fric}}{\mathrm{fd}}_{p}^{3}$$ (1)

where the energy decomposition gives three energy contributions, from left to right, due to fracture propagation, elastic deformation, and frictional dissipation, respectively. The prefactors *k*_frac_, *k*_elas_, and *k*_fric_ are related to the slenderness (length-to-base ratio) of the puncture tool and the stretchability of the target material (*36*, *37*), while Γ, μ, and *f* denote the fracture toughness/critical strain energy release rate, shear modulus, and frictional constant (*36*), respectively.

In contrast to a linear puncture tool, the deep penetration of a curved puncture tool exhibits unique characteristics such as a changing tool orientation with increasing distance *d_p_*. We define the angle of attack for an arbitrary cross section perpendicular to the centerline, α, as the angle between the local velocity vector and the target material surface following the convention in classic wound ballistics (*38*). For a linear puncture tool, as shown in Fig. 2, the angle of attack has a constant radian value of α = π*/*2 throughout the tool length. However, the angle of attack of a curved puncture tool increases linearly along the centerline with an increasing distance of *s* measured from the tip (Fig. 2, A and B). The minimum and maximum angle of attack are found to be α = π/2 − *d_p_*/ρ at the tip and α = π/2 at the target material surface, respectively. For an arbitrary cross section of interest at *s*, the local angle of attack is calculated by α = π/2 − (*d_p_* − *s*)/ρ. In Fig. 2, we take an infinitesimally thin slice, *ds* = ρ*d*θ, at this cross section and establish a local polar coordinate system, (*r,*θ), with the origin located at the rotational center. It can be shown that under such a coordinate transformation, the effect of different local angles of attack is irrelevant in a homogeneous and isotropic target material. Therefore, for a sufficiently small degree of curvature Θ (or a large radius of curvature of ρ relative to the tool length *L*), as observed in biological puncture tools (Θ ≈ 0° to 137°, Fig. 1) and demonstrated in Fig. 4, the mechanical interactions between the curved tool and target material within the thin slice *ds* → 0 are mathematically identical to those of a linear puncture tool, as shown by the comparison between Fig. 2C and Fig. 2D. This primarily stems from the geometric similarities between the curved and linear puncture tools and the orientation independence of the target material’s properties. Therefore, the energy contributions within the thin slice *ds* can be approximated by (∂*W*(*s*)/∂*s*)*ds* for both curved and linear puncture tools, where *W*(*s*) is determined from Eq. 1 with substitution. The total required energy for deep penetration takes an identical curvature-independent form corresponding to Eq. 1 after integration. This invariability of the puncture energetics is further supported by the FE scaling relationships in Fig. 4G.

Last, we speculate that the above theory may require additional considerations for curved puncture tools having an extremely large degree of curvature or ratio of length and radius of curvature (i.e., Θ = *L*/ρ). For a fixed tool length of *L*, a considerably smaller radius of curvature (ρ ≪ *L*) may cause self-interference of the elastic deformation field on the concave side of the curved puncture tool. The effect of such self-interference is likely not mathematically separable via simple superimposition in a nonlinear material, which can consequently alter the boundary conditions of the expansion of the puncture cavity (*36*) and thus the magnitude of the total stored elastic energy. However, we do not expect such curvature-induced disturbance of puncture energetics to occur within a biologically relevant range of morphology (e.g., Θ ≈ 0° to 137°, Fig. 1), as supported by our FE simulation results in Fig. 4 up to an extreme curvature of Θ = 180°.

### Effect of angle of attack on puncture damage

A core hypothesis of the above theory is that the damage created by deep penetration does not depend on the applied angle of attack in a homogeneous and isotropic target material. To provide further experimental evidence for this hypothesis, we performed dynamic puncture tests with controlled angles of attacks using silicone samples with inclined target surfaces (Materials and Methods). Figure 2J illustrates the resultant maximum depth of puncture produced at different applied angles of attack ranging from α = 40° to α = 90° and measured along the centerline of the undeformed fracture surface. As anticipated, the effect of angle of attack on the extent of puncture damage is insignificant within the tested range of variations, as verified by one-way analysis of variance (ANOVA) (*P* = 0*.*97). However, we note that further reducing the applied angle of attack will result in the inclined angle of the target surface approaching or becoming even smaller than the half cusp angle of the conical puncture tool. In this case, the contact between the conical tool surface and the target surface occurs first, and the induced shearing and friction need to be taken into account, which may deflect the puncture tool before a successful penetration. Such a special scenario is less relevant to the deep penetration of a curved puncture tool but may occur in and be leveraged by natural defensive mechanisms such as shells and armors with inclined surfaces (*39*, *40*).

### Effect of curvature on damage initiation

Force versus displacement responses for low-rate damage initiation tests on Sol-1:1 and Syl-20:1 target materials were obtained using various tool curvatures (Θ = [0*,* 20*,* 40*,* 60*,* 80]°), with all raw data presented in figs. S1 and S2. Consistent across tested conditions, force *F* increases monotonically with displacement *d* and then sharply drops at a critical point (open red circles) before increasing with *d* again. This critical point indicates the onset of puncture damage initiation, marking a key phase shift from elastic indentation to damage propagation. Notably, force and displacement data during elastic indentation of Sol-1:1 samples, when normalized by corresponding critical values (*F_c_* and *d_c_*) at the damage initiation, respectively, collapse into a single master curve (Fig. 3C). This behavior indicates the invariance and curvature independence of the contact mechanics governing the elastic indentation phase up to damage initiation.

We further evaluated the effect of tool curvature on damage initiation by analyzing the critical force (*F_c_*), critical displacement (*d_c_*), and work to initiate puncture (*W_c_*) from low-rate tests on Sol-1:1 and Syl-20:1 target materials. These metrics were derived from the force-displacement curves, with *W_c_* calculated as the area under the curve up to *d_c_* (Fig. 3C). The results are summarized in Fig. 3 (D to I) as box-and-whisker plots across different applied degrees of curvature (Θ). A one-way ANOVA was performed for each dataset to quantify the dependence on Θ. On the basis of the resultant *F* values and *P* values in table S1, there is no statistically significant impact of tool curvature on *F_c_* or *d_c_* (*P >* 0*.*05) in either silicone target materials within our tested range of Θ. Similarly, tool curvature does not significantly affect *W_c_* in Sol-1:1 (*P* = 0*.*30) and shows marginal significance in Syl20:1 (*P* = 0*.*044), although no individual group differences are statistically significant in post hoc comparisons (*P >* 0*.*05). The slight increase in *W_c_* at Θ = 80° for Syl-20:1 was likely caused by more stable and aggregated critical points due to the lack of early damage initiation in the replicate tests (see fig. S2).

The above analysis shows that the tool’s structural curvature has a minimal impact on damage initiation within a biologically relevant range of variation. Despite this finding being obtained at a low rate, we theorize its generalization to dynamic puncture scenarios within a broader range of puncture rates. This is because the local critical stress and deformation state associated with the initiation of puncture fracture are primarily governed by the intrinsic toughness and rate-dependent properties in soft elastic materials (*41*), which determine the critical force and displacement given specific contact geometries between the puncture tool tip and target material surface. The uniformity in the tip region across our curved puncture tools, including a similar shape, radius (*r* ≈ 75 μm), and perpendicular configuration, supports the assumption of consistent contact mechanics during elastic indentation and at damage initiation, leading to a similar contact area, critical load, and deformation irrespective of tool curvature. Consequently, other nonlocal curvature-related effects, such as a bending moment of *F*ρ(1 − cosΘ) induced by the eccentric reaction force, *F*, at the tool tip (Fig. 3B), is unlikely to affect elastic indentation and the critical conditions at damage initiation. Such a bending moment can potentially result in a horizontal scratching force component acting on the target surface as a preload. However, Fig. 3 (D and G) shows no evidence of a substantial change in the critical force in the puncture direction. Moreover, we recognize that the measured critical displacements at damage initiation in Fig. 3 (E and H) are primarily contributed by the elastic indentation of the target material. In contrast, the deflection of the tool tip is negligible given the considerably higher stiffness of the tool material (*E* ~ 3 GPa, clear resin, technical data sheet, Formlabs Inc.) compared to the target material [*E* ~ 0*.*4 MPa (*42*, *43*)]. Therefore, the corresponding critical puncture force is primarily governed by the contact mechanics rather than the bending stiffness of the puncture tool—the latter can vary markedly with tool curvatures, leading to different reaction forces when a fixed tip deflection/displacement is applied (*21*).

### Effect of curvature on the energetics of deep penetration

Figure 4E illustrates the simulated undeformed puncture damage/fracture surfaces for different structural curvatures (Θ = [0*,* 20*,* 40*,* 60*,* 80*,* 180]°) in orange against the blue target material matrix in the *x*-*y* plane. The damage near the material surface exhibits similarity and aligns vertically across all curvatures due to the tool’s predefined perpendicular configuration (Fig. 4D) and circular path. However, at deeper distances of penetration, the damage morphology increasingly deviates from the initial vertical direction with increasing tool curvature (Fig. 4E). As a result, while each curved puncture tool travels the same total distance *d*_*p,*max_ within the target material, the extent of puncture damage in the vertical direction diminishes as tool curvature increases. Such reduction of the vertical extent is estimated to be less than 4% at Θ = 80°, and approximately 17% at Θ = 180°, compared to a linear tool at *d*_*p,*max_ = 5 mm. A sufficiently large vertical extent relative to the tool length can be critical for venom-injecting organisms, such as vipers, spiders, and scorpions, which rely on the depth and accuracy of the deep penetration of their fangs or stingers to effectively deliver venom into the subcutaneous tissue or bloodstream of their prey (*4*, *13*, *19*). However, we expect the impact of curvature on the effectiveness of venom injection to be minor in these natural puncture systems, considering some of the most curved venom-injecting elements to our knowledge (Fig. 1) (e.g., the stinger of an Asian forest scorpion, with Θ ≈ 116° and an estimated decrease in vertical extent of ≈7% compared to a linear tool) and the relative location of the venom pore (*4*, *13*, *19*). In addition, the width of the puncture damage narrows along its contour, similar to the tapering of the triangular-shaped damage observed with a linear tool (Fig. 4E). Furthermore, the von Mises stress field near the fully deformed puncture cavity at the maximum distance of penetration exhibits similar magnitude and extent across different curvatures, as shown in Fig. 4F, suggesting comparable elastic contributions despite variations in local contours.

The relative magnitude of the energies necessary for deep penetration is presented in the bar plot in Fig. 4H for different curvatures. A puncture coefficient of efficiency, η = *W*_frac_/(*W*_frac_ + *U*_elas_), is also provided in Fig. 4H, indicating the fraction of the total energy used for creating puncture damage. Consistent with the results of damage initiation, there is no systematic effect of structural curvature on the energy parameters of deep penetration within a biologically relevant range of variation (Θ = 0° to 80°). The overall normalized root mean square error between the resultant energy parameters and their average values remains *<*5%. The small differences between the relative magnitudes of *W*_frac_ can be attributed to the limited resolution of the FE contact model at large curvatures. Furthermore, our additional simulation at Θ = 180° extends this finding to an extremely curved tool shape. To our knowledge, the largest naturally occurring tool curvature belongs to raptor talons with Θ ~ 140° (Fig. 1). A degree of curvature beyond this value is less likely to occur in biological puncture tools but will be relevant to biomedical applications such as 1/2 circle suture needles (*32*).

Further evidence of the independence of deep penetration on the effect of curvature can be obtained from the closely overlapping scaling relationships between normalized energies and distance of penetration extracted from the full history of deep penetration simulations across different curvatures, as illustrated in Fig. 4G. The negligible curvature effect on the scaling relationship is anticipated considering (i) the similar magnitude of energies at *d*_*p,*max_ and (ii) the self-similar configuration throughout the propagation of a curved tool. The stored elastic energy and the work to fracture are normalized by the shear modulus and the maximum distance of penetration using the expressions, *U*_elas_/μ*d*^3^_*p,*max_ and *W*_frac_/μ*d*^3^_*p,*max_, respectively. The best power-law fits of the energy data within a range of *d_p_*/*d*_*p,*max_ = 0*.*2 to 1 return close exponent values across Θ = 0° to 180°, with mean and SD, *n* = 2*.*5 ± 0*.*05, for the scaling relationship between normalized elastic energy *U*_elas_/μ*d*^3^_*p,*max_ and *d_p_*/*d*_*p,*max_, and *n* = 2*.*1 ± 0*.*08 between normalized work to fracture *W*_frac_/μ*d*^3^_*p,*max_ and *d_p_*/*d*_*p,*max_. Because the energy decomposition of a linear tool is well defined by Eq. 1, these coinciding power-law master curves indicate a curvature-independent energy-distance scaling relationship (i.e., Eq. 1) governing the energetics of deep penetration. On a side note, the power-law fitting for elastic energy in Fig. 4G accounts for a finite contribution from elastic deformation near the spherical tool tip region, which corresponds to the initial deviation of the FE scaling relationships at *d_p_*/*d*_*p,*max_ *<* 0*.*2 and likely contributes to the discrepancy between the best-fit exponent result and the theoretical prediction, *U*_elas_/μ *~ d*^3^*_p_*. Furthermore, while frictional contribution is not included in our FE model, we expect a similar convergence of scaling relationships across different curvatures due to similar contact areas at a prescribed *d_p_* between curved and linear tools.

## DISCUSSION

Our integrated approach, combining puncture experiments, FE damage simulation, and energetic theory, yields negative results that are consistent with the null hypothesis that a finite structural curvature of a tapered puncture tool does not have a substantive impact on the mechanics of damage initiation or deep penetration during puncture within a wide biologically relevant curvature range. While such curvature independence is theoretically limited to isotropic and homogeneous target materials during deep penetration, we speculate that it generally holds for typical biological puncture scenarios. For instance, in an animal integumentary system featuring a thin outer skin-like layer (~1 mm) (*44*) covering a thicker subcutaneous layer of adipose tissues, puncture of the skin layer is likely dominated by damage initiation, which is governed by material properties and contact mechanics, and remains largely unaffected by curvature, as demonstrated in Fig. 3. Similarly, the effect of curvature on deep penetration of the isotropic and homogeneous adipose layer is negligible. However, anisotropic effects can become notable if penetration occurs in muscle layers or collagen-rich tissues with aligned microfibers (*45*), which may lead to directional variation in material properties and damage resistance or localized stress redistribution (*44*, *45*). As a result, depending on the alignment of these anisotropic properties with the curvature, curved puncture tools might influence the energetics and damage pattern differently than linear tools, necessitating further exploration of these complex mechanical interactions.

Broadly, findings from this work may have far-reaching implications for understanding the diversity of biological puncture. Curved tapered tools are prevalent in natural puncture organisms, exhibiting wide morphological variations and supporting diverse biological functions beyond just puncture. While the presence of curvature is often seen as a positive adaptation, the evolutionary origin and biomechanical advantages of such curvature remain to be fully understood. Our findings reveal that structural curvature plays a minimal role in the efficiency of biological puncture. An important implication of this counterintuitive conclusion is that there is minimal trade-off between the puncture performance and other key performance objectives in biological puncture systems related to morphological variations of structural curvature. Consequently, this release of mechanical constraint may allow organisms to maintain a higher level of diversification potential for structural curvature development and more freedom to evolve for (i) high structural integrity to prevent tool buckling and damage and (ii) enhanced capabilities in secondary biological functions such as gripping, scratching, pinching, and slashing. Future research may explore how curvature impacts and mediates the optimal trade-off between these performance objectives.

## MATERIALS AND METHODS

### Sample preparation

Two highly stretchable, neo-Hookean-like silicone elastomers were selected as soft tissue simulants to mimic the ductile failure behavior (*36*, *37*, *43*): Sol-1:1 (Solaris, SmoothOn Inc.) with a 1:1 mixing ratio (w:w, part A to part B) and Syl-20:1 (Sylgard 184, Dow Corning) with a 20:1 mixing ratio (w:w, prepolymer base to curing agent). Samples were fabricated following documented procedures in (*35*, *37*), respectively. Each cubic silicone sample was standardized at approximately 49 mm × 49 mm × 42 mm (*t* × *w* × *h*) with a weight of approximately 100 ± 1 g.

Silicone samples featuring an inclined surface were fabricated by positioning a bisector (a thin polycarbonate sheet) vertically inside a cubic sample mold at a designed angle before pouring the liquid polymer mixture. After curing, the larger trapezoid sample was extracted for testing. The nominal angle (α) between the inclined sample surface and the horizontal plane varies between α = 40° and α = 90° in 10° increments.

### Puncture tool modeling

Figure 1 presents a simplified model of a biological puncture tool having a finite structural curvature. It is depicted as a curved cone, similar to the geometry of naturally occurring puncture elements as described in the literature (*6*, *21*). The centerline of the curved puncture tool is a circular arc having a controlled arc length, *L* ≈ 15 mm. Unless otherwise specified, the degree of curvature of the centerline, Θ = *L*/ρ, varies within a range, Θ = [0*,* 20*,* 40*,* 60*,* 80]°, where ρ is the radius of curvature. The centerline resides within the *x*-*y* plane. At the base of the puncture tool, the tangent to the centerline is perpendicular to the *x* axis and parallel to the *y* axis (Fig. 1). Any arbitrary cross section of the tool, taken perpendicular to the centerline, has a circular shape, with its diameter linearly diminishing from *D*_0_ at the base to *D_t_* at the tip along the centerline. The tip of the puncture tool is modeled as a hemisphere with a predetermined diameter of *D_t_* = 0*.*15 mm. The length-to-base ratio, LBR ≈ (*L* + 0*.*5*D_t_*)/*D*_0_, defines the slenderness or body sharpness of the puncture tool and is set as a controlled variable (LBR = 6) for all curved tool geometries examined in this work.

### Low-rate puncture method

Low-rate tests were performed on the two silicone simulant materials using a universal test stand (Instron 5944, Instron Inc.) to characterize damage initiation. Five different puncture tools, with degrees of curvature Θ = [0*,* 20*,* 40*,* 60*,* 80]° (including a linear tool), were fabricated using a stereolithography three-dimensional (3D) printer (Form 3, Formlabs Inc., clear resin, FLGPCL04, resolution: 25 μm). These curved tools were upscaled by a factor of 2 from the original geometrical model. The base of each puncture tool was affixed to a thin mounting plate to create a single component. Upon mounting, the center of the tool base was aligned with the centerline of the crosshead, and the tool tip was positioned perpendicular to the target surface of a cubic silicone sample (Fig. 3) placed on a compression stage. During each damage initiation test, the puncture tool indented the target surface at a rate of 10 mm/min until reaching a designed maximum displacement (6 mm for Sol-1,1 and 8 mm for Syl-20:1), ensuring penetration of the target surface within the displacement range. The critical displacement and force at the onset of damage initiation and the work to initiate were calculated and recorded. At least five replicate experiments were performed for each combination of material and tool curvature.

### Dynamic puncture method

Dynamic puncture tests for the effect of angle of attack on deep penetration were conducted in silicone samples with an inclined surface using an established experimental method (*35*–*37*). A customized compressed air cannon (Ballistic Loading and Structural Testing Lab, North Carolina State University) was used. 3D-printed conical projectiles (Form 3, Formlabs Inc., clear resin, FLGPCL04, resolution: 25 μm) having a designed cusp angle (30°) and tip radius (≈96 ± 5 μm) were selected as puncture tools (Fig. 2, G to I). The conical surface of the puncture tools was polished with 1500-grit sandpaper. During each dynamic puncture test, a sample was positioned with its inclined surface opposing the puncture direction, as illustrated in Fig. 2G. The puncture tool’s angle of attack (α) matched the angle between the inclined surface and the horizontal plane, which ranges from 40° to 90°. A controlled dynamic puncture speed, *v* = 10*.*3 ± 0*.*3 m/s (mean and SD), was maintained throughout all tests. The speed was selected considering the lowest stable speed producible by the air cannon. Speed calibration was performed using high-speed imaging (FASTCAM SA-Z, Photron Inc., 10,000 frames/s) immediately before the puncture tool impacted the inclined surface (Fig. 2H).

### FE simulations

A 3D FE damage model, as shown in Fig. 4, was implemented in Abaqus/Explicit to simulate fracture propagation, stress field, and energetics associated with deep penetration of curved puncture tools. Puncture tools with different curvatures were modeled as discrete rigid parts following Puncture tool modeling, including an extremely curved tool with Θ = 180°. The tip of each puncture tool was in contact and perpendicular to the target material surface in the initial configuration. The target material was modeled as a cylindrical space (Fig. 4A) and adopted an incompressible neo-Hookean model with ultimate properties corresponding to those of Sol-1:1 (*36*). The tool-material interaction during penetration adopted a frictionless contact condition (*36*). To simulate a realistic and biologically relevant deep penetration scenario, we assumed that the puncture tool’s curvature dictates its circular motion or rotation within the target material, as evidenced by high-speed imaging in naturally occurring puncture scenarios such as biting and stinging (*4*, *11*). A center of rotation coincident with the center of the circular centerline was defined for each puncture tool (Fig. 4, A and B), with a constant angular velocity corresponding to an effective feeding speed of 0.5 m/s applied. This ensured that any arbitrary point on the puncture tool maintained a displacement/velocity vector tangential to the centerline and perpendicular to its cross section throughout deep penetration. The simulation was carried out until the tool tip had traveled a total distance of *d*_*p,*max_ = 5 mm along the centerline. The resultant energetic parameters, such as elemental stored and dissipated strain energy density, were extracted from the model and integrated to estimate the energies required for puncture at the maximum distance of penetration *d*_*p,*max_. These energy components, similar to those identified with a linear conical tool (Eq. 1), stem from a decomposition of the total energy investment/external work done for deep penetration, which includes the dissipated energy for creating fracture surfaces (*W*_frac_), stored elastic energy (*U*_elas_) due to deformation to accommodate the tool shape, and frictional dissipation (which is equal to zero in our simplified “frictionless” FE model). The relative magnitudes of *W*_frac_ and *U*_elas_ across Θ = [0*,* 20*,* 40*,* 60*,* 80*,* 180]° were calculated with respect to their average values. Other technical details of FE modeling, including the substrate dimensions, mesh size, constitutive model, and energy-based failure criterion, are presented in Fig. 4 or documented in (*36*).

## Supplementary Materials

### This PDF file includes:

Figs. S1 and S2 Table S1 Legend for table S2 References
