# Supplementary material for: Curving expectations: The minimal impact of structural curvature in biological puncture mechanics
Source: Sci Adv. 2024 Aug 14;10(33):eadp8157. doi: 10.1126/sciadv.adp8157 (PMC11323891; doi:10.1126/sciadv.adp8157)
Supplement: Supplementary file 1 — Figs. S1 and S2 Table S1 Legend for table S2 References [file sciadv.adp8157_sm.pdf]

Science Advances
AAAS

Supplementary Materials for

Curving expectations: The minimal impact of structural curvature in biological puncture mechanics

Bingyang Zhang *et al.*

Corresponding author: Bingyang Zhang, bzhang53@illinois.edu; Philip S. L. Anderson, andersps@illinois.edu

Sci. Adv. **10**, eadp8157 (2024)
DOI: 10.1126/sciadv.adp8157

The PDF file includes:

Figs. S1 and S2
Table S1
Legend for table S2
References

Other Supplementary Material for this manuscript includes the following:

Table S2

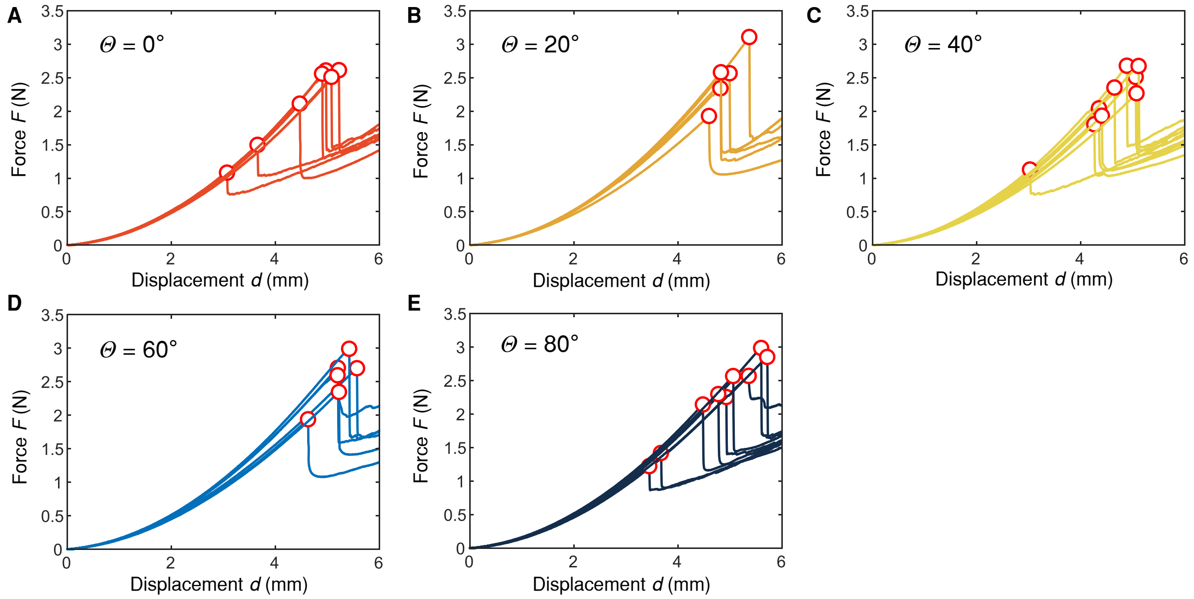

Fig. S1.

Force vs. displacement curves for all damage initiation tests of Solaris 1:1 samples, grouped by applied degree of curvature (Θ = [0, 20, 40, 60, 80]°). Orange open circles indicate the critical points of damage initiation.

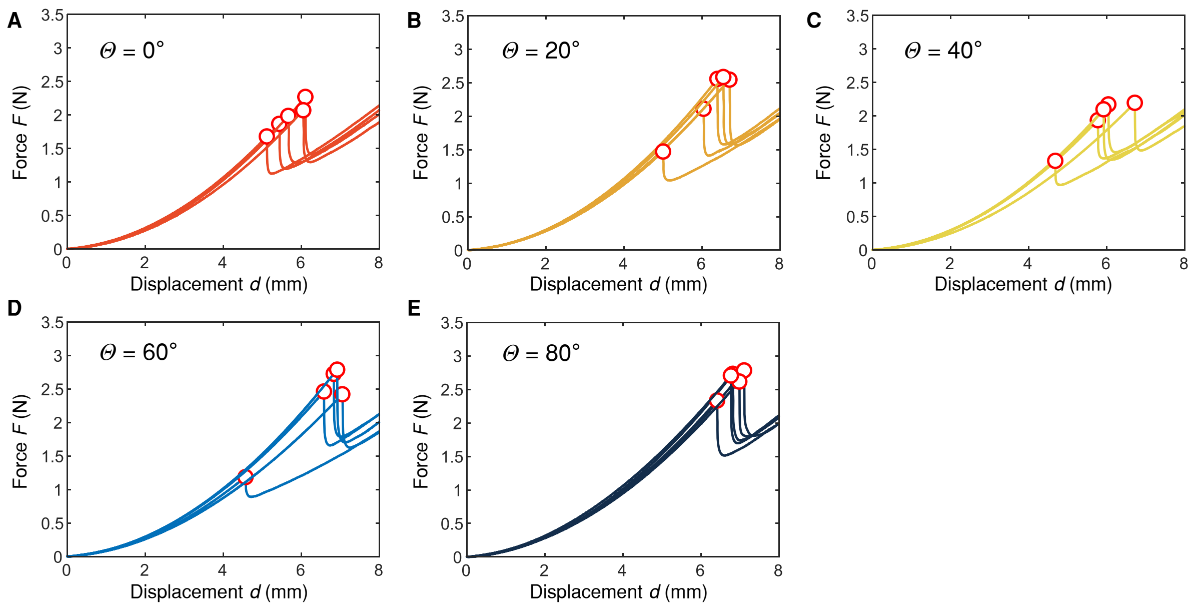

Fig. S2.

Force vs. displacement curves for all damage initiation tests of Sylgard 20:1 samples, grouped by applied degree of curvature (Θ = [0, 20, 40, 60, 80]°). Orange open circles indicate the critical points of damage initiation.

Critical parameters	Sol-1:1			Syl-20:1		
	F_c	d_c	W_c	F_c	d_c	W_c
F-value	0.88	1.34	1.28	2.29	2.24	2.99
p-value	0.49	0.28	0.30	0.095	0.1	0.044

Table S1.

Supplementary Table 1: Results of one-way ANOVA for the influence of curvature on critical parameters at damage initiation of PDMS target materials.

Table S2.

Supplementary Table 2: Structural curvature measurements of different biological puncture tools. See the separate spreadsheet for details.

REFERENCES AND NOTES

1. D. W. Fowler, E. A. Freedman, J. B. Scannella, Predatory functional morphology in raptors: Interdigital variation in talon size is related to prey restraint and immobilisation technique. *PLOS ONE* **4**, e7999 (2009).

2. M. Halpern, A. Waissler, A. Dror, S. Lev-Yadun, Biological warfare of the spiny plant, in *Advances in Applied Microbiology* (Elsevier Inc., ed. 1, 2011), vol. 74, pp. 97–116.

3. F. Gallenmüller, A. Feus, K. Fiedler, T. Speck, Rose prickles and asparagus spines—Different hook structures as attachment devices in climbing plants. *PLOS ONE* **10**, e0143850 (2015).

4. Z. L. Zhao, T. Shu, X. Q. Feng, Study of biomechanical, anatomical, and physiological properties of scorpion stingers for developing biomimetic materials. *Mater. Sci. Eng. C* **58**, 1112–1121 (2016).

5. A. van der Meijden, T. Kleinteich, A biomechanical view on stinger diversity in scorpions. *J. Anat.* **230**, 497–509 (2017).

6. B. Bar-On, On the form and bio-mechanics of venom-injection elements. *Acta Biomater.* **85**, 263–271 (2019).

7. P. S. L. Anderson, Making a point: Shared mechanics underlying the diversity of biological puncture. *J. Exp. Biol.* **221**, jeb187294 (2018).

8. B. Bhushan, Insects locomotion, piercing, sucking and stinging mechanisms. *Microsyst. Technol.* **24**, 4703–4728 (2018).

9. S. B. Crofts, Y. Lai, Y. Hu, P. S. L. Anderson, How do morphological sharpness measures relate to puncture performance in viperid snake fangs? *Biol. Lett.* **15**, 20180905 (2019).

10. K. H. Jensen, J. Knoblauch, A. H. Christensen, K. S. Haaning, K. Park, Universal elastic mechanism for stinger design. *Nat. Phys.* **16**, 1074–1078 (2020).

11. W. G. Ryerson, T. Van Valkenburg, Linking tooth shape to strike mechanics in the boa constrictor. *Integr. Comp. Biol.* **61**, 759–771 (2021).

12. T. I. Pollock, D. P. Hocking, A. R. Evans, The killer's toolkit: Remarkable adaptations in the canine teeth of mammalian carnivores. *Zool. J. Linn. Soc.* **196**, 1138–1155 (2022).

13. S. B. Crofts, P. S. Anderson, How venom pore placement may influence puncture performance in snake fangs. *J. Exp. Biol.* **226** jeb245666 (2023).

14. H. Quan, X. Liang, X. Zhang, M. A. Meyers, R. M. McMeeking, E. Arzt, The shape of nature's stingers revealed. *Proc. Natl. Acad. Sci. U.S.A.* **121**, e2316320121 2017 (2024).

15. W. K. Cho, J. A. Ankrum, D. Guo, S. A. Chester, S. Y. Yang, A. Kashyap, G. A. Campbell, R. J. Wood, R. K. Rijal, R. Karnik, R. Langer, J. M. Karp, Microstructured barbs on the North American porcupine quill enable easy tissue penetration and difficult removal. *Proc. Natl. Acad. Sci. U.S.A.* **109**, 21289–21294 (2012).

16. Z.-L. Zhao, H.-P. Zhao, G.-J. Ma, C.-W. Wu, K. Yang, X.-Q. Feng, Structures, properties, and functions of the stings of honey bees and paper wasps: A comparative study. *Biol. Open* **4**, 921–928 (2015).

17. C. S. Shea-Vantine, K. A. Galloway, D. N. Ingle, M. E. Porter, S. M. Kajiura, Caudal spine morphology and puncture performance of two coastal stingrays. *Integr. Comp. Biol.* **61**, 749–758 (2021).

18. S. B. Crofts, P. S. L. Anderson, The influence of cactus spine surface structure on puncture performance and anchoring ability is tuned for ecology. *Proc. Biol. Sci.* **285**, 20182280 (2018).

19. Y. Politi, M. Priewasser, E. Pippel, P. Zaslansky, J. Hartmann, S. Siegel, C. Li, F. G. Barth, P. Fratzl, A spider's fang: How to design an injection needle using chitin-based composite material. *Adv. Funct. Mater.* **22**, 2519–2528 (2012).

20. U. Cerkvenik, B. van de Straat, S. W. S. Gussekloo, J. L. van Leeuwen, Mechanisms of ovipositor insertion and steering of a parasitic wasp. *Proc. Natl. Acad. Sci. U.S.A.* **114**, E7822–E7831 (2017).

21. B. Bar-On, The effect of structural curvature on the load-bearing characteristics of biomechanical elements. *J. Mech. Behav. Biomed. Mater.* **138**, 105569 (2023).

22. R. M. S. Schofield, J. Bailey, J. J. Coon, A. Devaraj, R. W. Garrett, M. S. Goggans, M. G. Hebner, B. S. Lee, D. Lee, N. Lovern, S. Ober-Singleton, N. Saephan, V. R. Seagal, D. M. Silver, H. E. Som, J. Twitchell, X. Wang, J. S. Zima, M. H. Nesson, The homogenous alternative to biomineralization: Zn- and Mn-rich materials enable sharp organismal "tools" that reduce force requirements. *Sci. Rep.* **11**, 17481 (2021).

23. B. Bar-On, F. G. Barth, P. Fratzl, Y. Politi, Multiscale structural gradients enhance the biomechanical functionality of the spider fang. *Nat. Commun.* **5**, 3894 (2014).

24. M. Tadayon, O. Younes-Metzler, Y. Shelef, P. Zaslansky, A. Rechels, A. Berner, E. Zolotoyabko, F. G. Barth, P. Fratzl, B. Bar-On, Y. Politi, Adaptations for wear resistance and damage resilience: Micromechanics of spider cuticular "tools". *Adv. Funct. Mater.* **30**, 2000400 (2020).

25. Y. Politi, L. Bertinetti, P. Fratzl, F. G. Barth, The spider cuticle: A remarkable material toolbox for functional diversity. *Philos. Trans. A. Math. Phys. Eng. Sci.* **379**, 20200332 (2021).

26 .R. Petie, M. Muller, Curvature facilitates prey fixation in predatory insect claws. *J. Theor. Biol.* **244**, 565–575 (2007).

27. M. S. DeVries, E. A. K. Murphy, S. N. Patek, Strike mechanics of an ambush predator: The spearing mantis shrimp. *J. Exp. Biol.* **215**, 4374–4384 (2012).

28. Y. Song, Z. Dai, Z. Wang, A. Ji, S. N. Gorb, The synergy between the insectinspired claws and adhesive pads increases the attachment ability on various rough surfaces. *Sci. Rep.* **6**, 1–9 (2016).

29. J. G. Pattrick, D. Labonte, W. Federle, Scaling of claw sharpness: Mechanical constraints reduce attachment performance in larger insects. *J. Exp. Biol.* **221**, jeb188391 (2018).

30. L. R. Tsang, L. A. B. Wilson, J. Ledogar, S. Wroe, M. Attard, G. Sansalone, Raptor talon shape and biomechanical performance are controlled by relative prey size but not by allometry. *Sci. Rep.* **9**, 7076 (2019).

31. L. Jeffries, D. Lentink, Design principles and function of mechanical fasteners in nature and technology. *Appl. Mech. Rev.* **72**, 1–24 (2020).

32. X. Bao, W. Li, M. Lu, Z. Zhou, Experiment study on puncture force between mis suture needle and soft tissue. *Biosurf. Biotribol.* **2**, 49–58 (2016).

33. A. Majewicz, S. P. Marra, M. G. van Vledder, M. Lin, M. A. Choti, D. Y. Song, A. M. Okamura, Behavior of tip-steerable needles in ex vivo and in vivo tissue. *I.E.E.E. Trans. Biomed. Eng.* **59**, 2705–2715 (2012).

34. S. Misra, K. B. Reed, B. W. Schafer, K. T. Ramesh, A. M. Okamura, Mechanics of flexible needles robotically steered through soft tissue. *Int. J. Rob. Res.* **29**, 1640–1660 (2010).

35. B. Zhang, P. S. L. Anderson, Investigation of the rate-mediated form-function relationship in biological puncture. *Sci. Rep.* **13**, 12097 (2023).

36. B. Zhang, P. S. L. Anderson, Modelling biological puncture: A mathematical framework for determining the energetics and scaling. *J. R. Soc. Interface* **19**, 20220559 (2022).

37. B. Zhang, P. S. Anderson, How rate-based stretchability of soft solids controls fracture morphology in dynamic conical puncture. *Int. J. Imp. Engine* **187**, 104911 (2024).

38. R. M. Coupland, M. A. Rothschild, M. J. Thali, *Wound Ballistics* (Springer Berlin Heidelberg, 2011).

39. M. J. Connors, H. Ehrlich, M. Hog, C. Godeffroy, S. Araya, I. Kallai, D. Gazit, M. Boyce, C. Ortiz, Three-dimensional structure of the shell plate assembly of the chiton tonicella marmorea and its biomechanical consequences. *J. Struct. Biol.* **177**, 314–328 (2012).

40. V. González-Albuixech, M. Rodríguez-Millán, T. Ito, J. Loya, M. Miguélez, Numerical analysis for design of bioinspired ceramic modular armors for ballistic protections. *Int. J. Damage Mech.* **28**, 815–837 (2019).

41. R. Long, C.-Y. Hui, Fracture toughness of hydrogels: Measurement and interpretation. *Soft Matter* **12**, 8069–8086 (2016).

42. B. Zhang, S. B. Hutchens, On the relationship between cutting and tearing in soft elastic solids. *Soft Matter* **17**, 6728–6741 (2021).

43. B. Zhang, "Y-shaped cutting as a characterization method for the failure of soft elastic solids," thesis, University of Illinois Urbana-Champaign (2021).

44. H. Joodaki, M. B. Panzer, Skin mechanical properties and modeling: A review. *Proc. Inst. Mech. Eng. H* **232**, 323–343 (2018).

45. K. Bircher, M. Zündel, M. Pensalfini, A. E. Ehret, E. Mazza, Tear resistance of soft collagenous tissues. *Nat. Commun.* **10**, 792 (2019).

46. A. J. Kohn, M. Nishi, B. Pernet, Snail spears and scimitars: A character analysis of conus radular teeth. *J. Moll. Stud.* **65**, 461–481 (1999).

47. R. Gal, M. Kaiser, G. Haspel, F. Libersat, Sensory arsenal on the stinger of the parasitoid jewel wasp and its possible role in identifying cockroach brains. *PLOS ONE* **9**, 1–10 (2014).

48. P. Christiansen, Canine morphology in the larger felidae: Implications for feeding ecology. *Biol. J. Linn. Soc.* **91**, 573–592 (2007).

49. L. Kundanati, R. Guarino, M. Menegon, N. M. Pugno, Mechanics of snake biting: Experiments and modelling. *J. Mech. Behav. Biomed. Mater.* **112**, 1–10 (2020).

50. A. du Plessis, C. Broeckhoven, S. G. le Roux, Snake fangs: 3D morphological and mechanical analysis by microct, simulation, and physical compression testing. *GigaScience* **7**, 1–8 (2018).

51. C. Broeckhoven, A. du Plessis, Has snake fang evolution lost its bite? New insights from a structural mechanics viewpoint. *Biol. Lett.* **13**, 1–4 (2017).

52. A. Palci, A. R. H. LeBlanc, O. Panagiotopoulou, S. G. C. Cleuren, H. Mehari Abraha, M. N. Hutchinson, A. R. Evans, M. W. Caldwell, M. S. Y. Lee, Plicidentine and the repeated origins of snake venom fangs. *Proc. R. Soc. B* **288**, 1–9 (2021).
